# The Neuronal PAS Domain Protein 4 (*Npas4*) Is Required for New and Reactivated Fear Memories

**DOI:** 10.1371/journal.pone.0023760

**Published:** 2011-08-22

**Authors:** Jonathan E. Ploski, Melissa S. Monsey, Tam Nguyen, Ralph J. DiLeone, Glenn E. Schafe

**Affiliations:** 1 School of Behavioral and Brain Sciences, University of Texas at Dallas, Richardson, Texas, United States of America; 2 Department of Psychology, Yale University, New Haven, Connecticut, United States of America; 3 Interdepartmental Neuroscience Program, Yale University, New Haven, Connecticut, United States of America; 4 Department of Psychiatry, Yale School of Medicine, New Haven, Connecticut, United States of America; Pontifical Catholic University of Rio Grande, Brazil

## Abstract

The Neuronal PAS domain protein 4 (Npas4) is a neuronal activity-dependent immediate early gene that has recently been identified as a transcription factor which regulates the transcription of genes that control inhibitory synapse development and synaptic plasticity. The role Npas4 in learning and memory, however, is currently unknown. Here, we systematically examine the role of Npas4 in auditory Pavlovian fear conditioning, an amygdala-dependent form of emotional learning. In our first series of experiments, we show that Npas4 mRNA and protein are regulated in the rat lateral nucleus of the amygdala (LA) in a learning-dependent manner. Further, knockdown of Npas4 protein in the LA via adeno-associated viral (AAV) mediated gene delivery of RNAi was observed to impair fear memory formation, while innate fear and the expression of fear memory were not affected. In our second series of experiments, we show that Npas4 protein is regulated in the LA by retrieval of an auditory fear memory and that knockdown of Npas4 in the LA impairs retention of a reactivated, but not a non-reactivated, fear memory. Collectively, our findings provide the first comprehensive look at the functional role of Npas4 in learning and memory.

## Introduction

The requirement of *de novo* transcription and translation is a hallmark of long-term memory formation [1]. Both vertebrate and invertebrate models of memory have emphasized the importance of the cAMP-response element (CRE) binding protein (CREB), a nuclear transcription factor which regulates the expression of genes that are thought to be critical for the functional and/or structural changes underlying long-term synaptic plasticity and memory [2], [3], [4]. However, while CREB and CRE-driven transcription have been consistently implicated in memory formation [4], the role of other transcription factors has received considerably less attention.

The neuronal PAS domain protein 4 (Npas4), also known as NXF, Le-PAS (Limbic Enriched PAS) and PASD10, is a basic helix-loop-helix (bHLH) transcription factor that is almost exclusively expressed in the brain and is enriched in limbic areas [5]. Npas4 contains a PAS domain, which is named after three proteins the domain is found in: Period circadian protein (Per), Aryl hydrocarbon receptor nuclear translocator protein (Arnt), and Single-minded protein (Sim). These domains are involved in protein-protein interactions that facilitate heterodimerization with other proteins to act as co-regulators of transcription. In addition, these domains can sometimes act as signal sensors binding ligands, which can alter their functioning [6], [7]. Npas4 is known to be regulated transcriptionally in response to seizure [8], cerebral ischemia [9], neural activity [10], restraint stress [11], and long-term potentiation [12], [13]. Further, Npas4 has recently been implicated in regulating a transcriptional program controlling homeostatic plasticity by inducing inhibitory synapse development [10]. At present, however, the functional role of Npas4 in cognitive functions such as learning and memory remains unknown.

In the present study, we examine the role of Npas4 in Pavlovian fear conditioning, an amygdala-dependent form of learning and memory. We show that both auditory fear conditioning and retrieval of an auditory fear memory regulate the expression of Npas4 in the lateral nucleus of the amygdala (LA). Further, viral-mediated knockdown of Npas4 in the LA impairs both fear memory formation and the retention of a reactivated fear memory. Collectively, our findings point to a vital role for Npas4 in both memory consolidation and reconsolidation processes in the mammalian brain.

## Results

### Npas4 is regulated in the LA by Pavlovian fear conditioning

**Figure 1:**
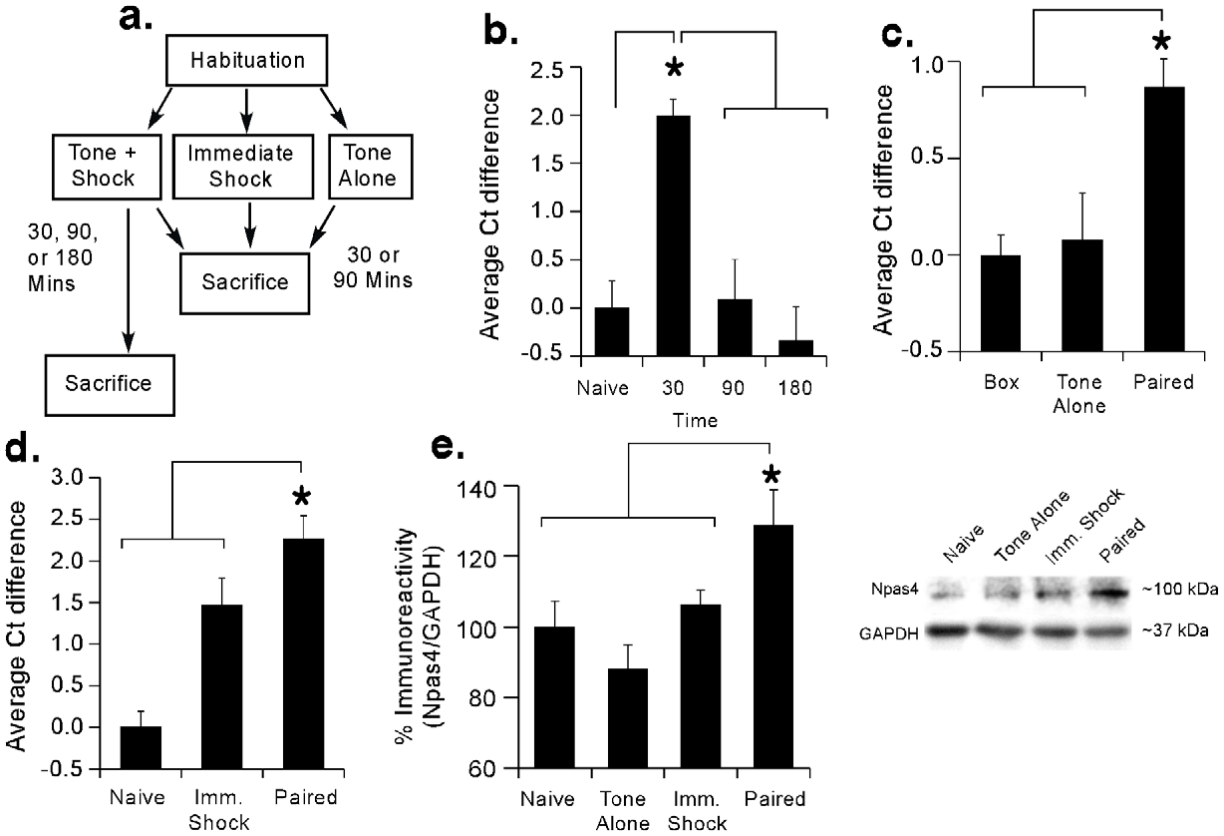
Regulation of Npas4 mRNA and protein in the LA following fear conditioning. (A) Schematic of the behavioral protocol for qRT-PCR and Western experiments. (B) Time course analysis of Npas4 mRNA expression in the LA following fear conditioning using qRT-PCR (n = 8/group). *p<0.05 relative to the other groups. (C) Regulation of Npas4 mRNA in the LA using qRT-PCR. Rats were sacrificed 30 min following exposure to fear conditioning (Paired; n = 8), tone alone (Tone Alone; n = 6) or the training environment alone (Box; n = 7). *p<0.05 relative to Box and Tone Alone groups. (D) Regulation of Npas4 mRNA in the LA using qRT-PCR. Rats were sacrificed 30 min following exposure to fear conditioning (Paired; n = 7), immediate shock (Imm. Shock; n = 8) or no stimulation (Naïve; n = 7). *p<0.05 relative to Naïve and Imm. Shock groups. (E) Western blot analysis of Npas4 protein in the LA. Rats were sacrificed 90 min following exposure to fear conditioning (Paired; n = 7), tone alone (Tone Alone; n = 7), immediate shock (Imm. Shock; n = 8) or no stimulation (Naïve; n = 8). *p<0.05 relative to the other groups. Representative Western blots are shown in the inset.

In our first series of experiments, we used qRT-PCR and Western blotting to examine the regulation of Npas4 mRNA and protein in the LA following auditory fear conditioning (Figure 1a). Rats were conditioned with tone-shock pairings and sacrificed either 30, 90, or 180 min after training. Relative to naïve controls, we observed a significant increase in Npas4 mRNA at 30 min following fear conditioning using qRT-PCR [Figure 1b; F(3,28)  = 11.32, p<0.001], with the 30 min group being significantly different from both naïve controls and the 90 and 180 min groups (p<0.05; Duncan's test). No significant differences were detected between the 90 and 180 min groups and naïve controls (p>0.05).

To examine whether training-induced regulation of Npas4 mRNA in the LA is specific to associative fear learning, we next used qRT-PCR and Western blotting to examine Npas4 in fear conditioned rats relative to conditions that do not support fear learning. In our first experiment, we observed an increase in Npas4 mRNA in fear conditioned rats relative to those exposed to tones without foot shocks or to the training chamber with no further stimulation [F(2,16)  = 8.15, p<0.01; Figure 1c], with the conditioned group being significantly different from both tone alone and box alone controls (p<0.05). The tone alone group did not differ from the box alone group (p>0.05). In a separate experiment, we observed increased Npas4 mRNA expression in trained rats relative to those exposed to immediate shocks of equivalent duration and intensity [F(2,19)  = 17.55, p<0.01; Figure 1d], with the conditioned group being significantly different from both immediate shock and naïve controls (p<0.05). The immediate shock group was also observed to differ from naïve controls (p<0.05), exhibiting an intermediate level of Npas4 mRNA expression between naive and conditioned rats. Finally, Western blotting showed that fear conditioning leads to a significant increase in Npas4 protein in the LA 90 min following training relative to naïve, tone alone, or immediate shock conditions [F(3,26)  = 5.37, p<0.01; Figure 1e], with the conditioned group being significantly different from each of the other groups (p<0.05). No significant differences were detected between groups exposed to immediate shock or tone alone and naïve controls (p>0.05).

### Npas4 is required for fear memory formation

**Figure 2:**
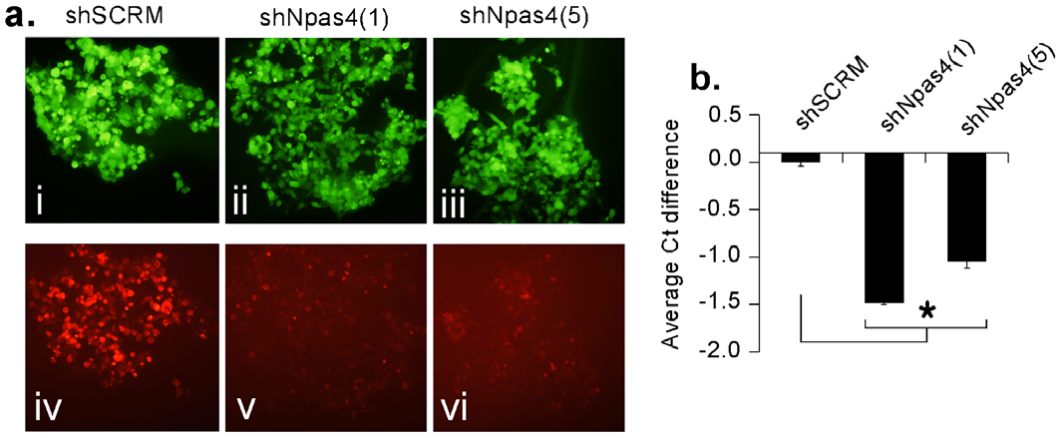
Knockdown of Npas4 *in vitro*. (A) HEK293 cells were co-transfected with plasmids expressing Npas4-RFP and plasmids expressing shRNAs designed to deplete Npas4 mRNA [shNpas4(1) (ii, v) and shNpas4(5) (iii, vi)]. A control shRNA (shSCRM) (i, iv) that does not target Npas4 was used as a control. Ninety-six hrs following transfection, cells were visualized by fluorescence microcopy (20X) for the presence of the shRNA GFP plasmids, (panels i, ii, iii) and Npas4-RFP (panels iv, v, and vi). Cells transfected with shNPAS4(1) and shNPAS4(5) exhibited significantly less Npas4-RFP compared to cells transfected with shSCRM, suggesting that the shRNAs against Npas4 are targeting the Npas4-RFP mRNA for degradation resulting in less Npas4-RFP protein (panels v. and vi. versus iv.). Exposure times were the same for visualization of GFP images and RFP images, respectively. (B) qRT-PCR analysis of Npas4 mRNA from samples prepared as described above. Both shNpas4(1) and shNpas4(5) significantly depleted Npas4 mRNA relative to the shSCRM control (n = 3, each group). p<0.05 relative to the shSCRM group.

To examine the functional role of Npas4 in fear memory formation, we next utilized a combination of adeno-associated viral (AAV) mediated gene delivery and RNA interference (RNAi) technology to deplete Npas4 from LA neurons prior to auditory fear conditioning. We first designed 5 different Npas4 specific short-hairpin RNAs (shRNA) and tested each for efficacy *in vitro* in HEK293 cells for Npas4 depletion. Cells were transfected with the plasmid dsRed1-N1 expressing rat Npas4 as a fusion protein with red fluorescent protein (RFP) and shNpas4 or shSCRM plasmids (Figure 2a). qRT-PCR was performed to detect changes in Npas4 expression between shRNA transfected cells vs. those transfected with an AAV vector expressing a scrambled shRNA (AAV-shSCRM). Two of the shRNAs, shNpas4(1) and shNpas4(5), were found to effectively deplete Npas4 *in vitro* [F(2,6)  = 255.5, p<0.01], with shNpas4(1) being slightly more effective (p<0.05; Duncan's test; Figure 2b).

#### Knockdown of Npas4 in the LA

**Figure 3:**
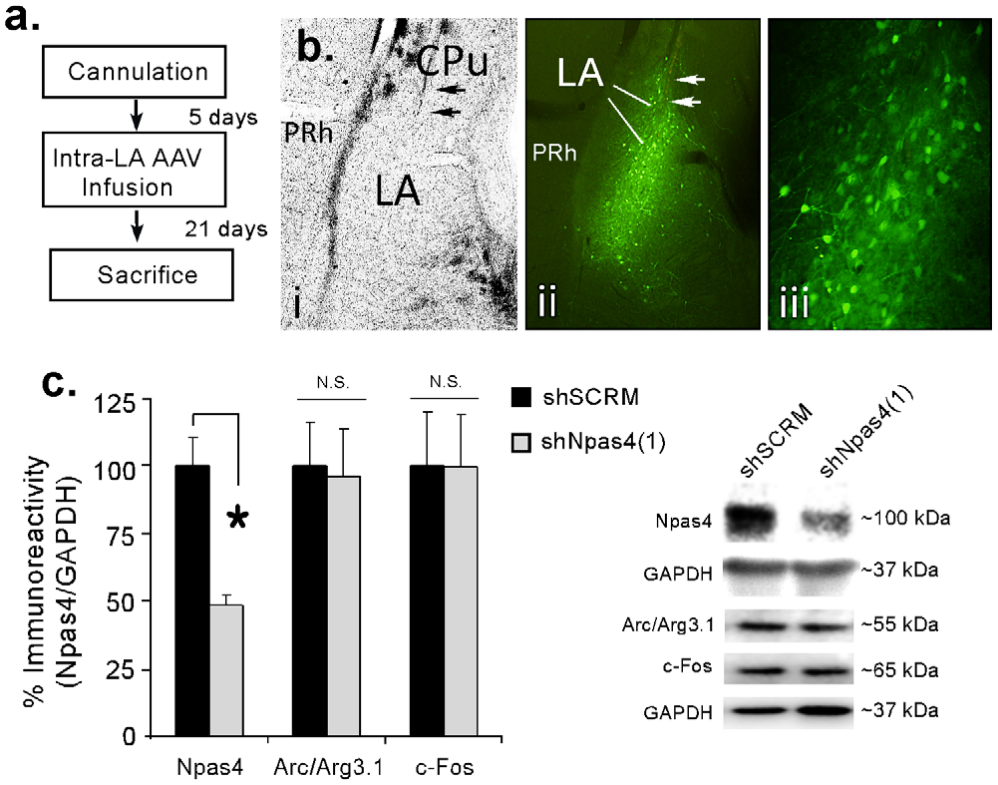
Knockdown of Npas4 in the LA. (A) Schematic of the experimental protocol. Rats were given intra-LA infusion of either AAV-shSCRM or AAV-ShNpas4 vectors and were sacrificed 21 days later. (B) Images depicting anatomical localization of AAV-infected cells expressing GFP and shNpas4(1) within the LA 21 days after infusion. (i) Region of the LA under bright field illumination. (ii) Same brain slice as depicted in (i), but under fluorescence to visualize GFP expressing neurons. (iii) Higher magnification image of transfected cells depicted in (ii). Arrows depict the infusion needle. (C) Analysis of Npas4, Arc/Arg3.1, and c-Fos proteins in LA homogenates taken 21 days following intra-LA infusion of AAV- shNpas4 (n = 9) or AAV-shSCRM (n = 8). *p<0.05 relative to AAV-shSCRM controls. Representative Western blots are depicted in the inset.

We next incorporated the most efficacious Npas4 shRNA, shNpas4(1), into AAV and tested for its ability to deplete Npas4 *in vivo*. Rats were given intra-LA infusion of AAV-shNpas4(1) (1 µl/side) or a scrambled control (AAV-shSCRM; 1 µl/side) and were sacrificed 21 days later (Figure 3a), a time point that has been shown to be optimal for shRNA expression and protein depletion *in vivo* [14]. Brain sections containing the LA were processed for fluorescence microscopy to visualize AAV infected neurons expressing GFP and shRNAs. There was a remarkable degree of infection and anatomical specificity (Figure 3b). GFP-expressing neurons were observed throughout the LA. The central nucleus of the amygdala (CE) and surrounding cortical regions did not exhibit significant infection. Additionally, infected neurons expressing shNpas4(1) or shSCRM did not exhibit any noticeable gross morphological changes or damage. Further, Western blotting revealed a significant decrease in Npas4 protein expression in LA homogenates taken from rats infused with AAV-shNpas4(1) relative to AAV-shSCRM controls [t(15)  = 4.75, p<0.01], indicating that Npas4 shRNA is effective at depleting Npas4 protein *in vivo* (Figure 3c). Importantly, we detected no significant changes in the immediate early genes (IEGs) Arc/Arg3.1 [t(15)  = 0.15, p>0.05] and c-Fos [t(15)  = 0.18, p>0.05] following infusion with shNpas4(1), suggesting that the AAV-shNpas4(1) vector is specific to Npas4.

#### Knockdown of Npas4 in the LA impairs fear memory formation, but not innate fear or fear expression

**Figure 4:**
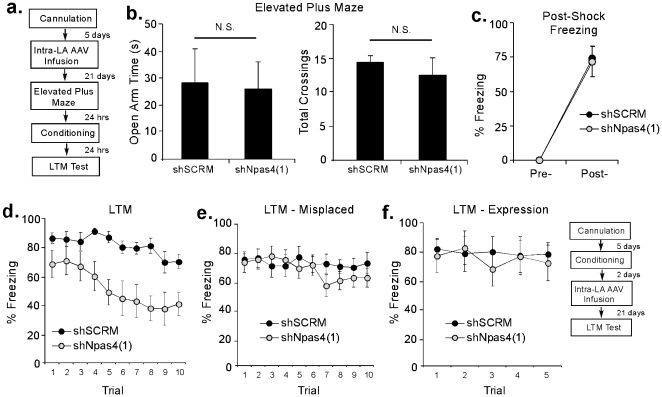
Knockdown of Npas4 in the LA impairs learned, but not innate, fear. (A) Schematic of the behavioral protocol. Rats were given intra-LA infusion of either AAV-shSCRM or AAV-ShNpas4 vectors followed by fear conditioning 22 days later. A portion of the rats was also tested in the elevated plus maze on the day prior to fear conditioning. (B) Analysis of open arm time (left) and total number of crossings (right) in AAV-shNpas4 (n = 4) and AAV-shSCRM (n = 7) groups in the elevated plus maze. (C) Post-shock freezing scores in AAV-shNpas4 (n = 10) and AAV-shSCRM (n = 10) groups immediately after the conditioning trial. (D) Auditory fear memory assessed at 24 hrs after fear conditioning in each group. (E) Auditory fear memory assessed at 24 hrs after fear conditioning in AAV-shNpas4 (n = 13) and AAV-shSCRM (n = 12) groups that had misplaced viral infection. (F) Expression of auditory fear memory in rats infused with AAV-shNpas4 (n = 7) or AAV-shSCRM (n = 7) following conditioning and tested for fear memory 21 days after viral infusion.

In behavioral experiments, we next examined the effect of Npas4 knockdown in the LA on both innate and learned fear. Rats were given intra-LA infusion of AAV-shNpas4(1) or AAV-shSCRM (1 µl/side) followed by auditory fear conditioning 22 days later consisting of a single presentation of a 20 sec, 5 kHz, 75 dB tone that co-terminated with a 1 sec, 2 mA foot shock (Figure 4a). A portion of the rats were next tested for innate fear in the elevated plus maze on the day prior to fear conditioning (Day 21; Figure 4a). Rats that received infusions of either AAV-shNpas4(1) or AAV-shSCRM exhibited no differences in open arm time or number of crossings in the elevated plus maze, indicating that Npas4 knockdown within the LA does not affect baseline innate fear responses [t(9) = 0.21, p>0.05] or locomotor behavior [t(9) = 0.84, p>0.05], respectively (Figure 4b).

Following auditory fear conditioning, both AAV-shNpas4(1) and AAV-shSCRM-infused rats exhibited intact post-shock freezing (Figure 4c), indicating that shock perception is not affected by Npas4 knockdown. The ANOVA revealed only a significant effect of trial (pre vs. post-training), F(1,18)  = 106.9 p<0.05. The effects for group [F(1,18)  = 0.02] and the group by trial interaction [F(1,18)  = 0.02] were not significant. When tested for retention of auditory fear memory 24 hr later, however, rats infused with AAV-shNpas4(1) exhibited impaired long-term memory (LTM) relative to AAV-shSCRM controls (Figure 4d). The ANOVA revealed a significant effect for group [F(1,18)  = 10.69, p<0.05], trial [F(9,162)  = 7.45, p<0.05], and the group by trial interaction [F(9,162)  = 1.96, p<0.05]. Further, rats with misplaced AAV-shNpas4 infusions failed to exhibit impaired LTM (Figure 4e). The ANOVA revealed no effects for group [F(1,23)  = 0.35], trial [F(9,207)  = 1.60], or the group by trial interaction [F(9,207)  = 1.12].

Next, we asked whether intra-LA infusion of AAV-shNpas4 affects the expression of an auditory fear memory. Rats were trained and given intra-LA infusion of AAV-shNpas4(1) or AAV-shSCRM 21 days prior to a test of auditory fear memory (Figure 4f, inset). No significant differences were observed between AAV-shNpas4(1) and AAV-shSCRM groups (Figure 4f). The ANOVA revealed no significant effect of group [F(1,12)  = 0.06], trial [F(4,48)  = 0.89], or the group by trial interaction [F(4,48)  = 1.03].

Thus, knockdown of Npas4 protein in the LA significantly impairs auditory fear memory formation, but has no significant effect on innate fear or the expression of auditory fear memory.

### Npas4 is required for retention of a reactivated fear memory

In a final series of experiments, we examined the role of Npas4 in the retention of a reactivated fear memory using a ‘reconsolidation’ paradigm [15].

#### Retrieval of a fear memory regulates Npas4 in the LA

**Figure 5:**
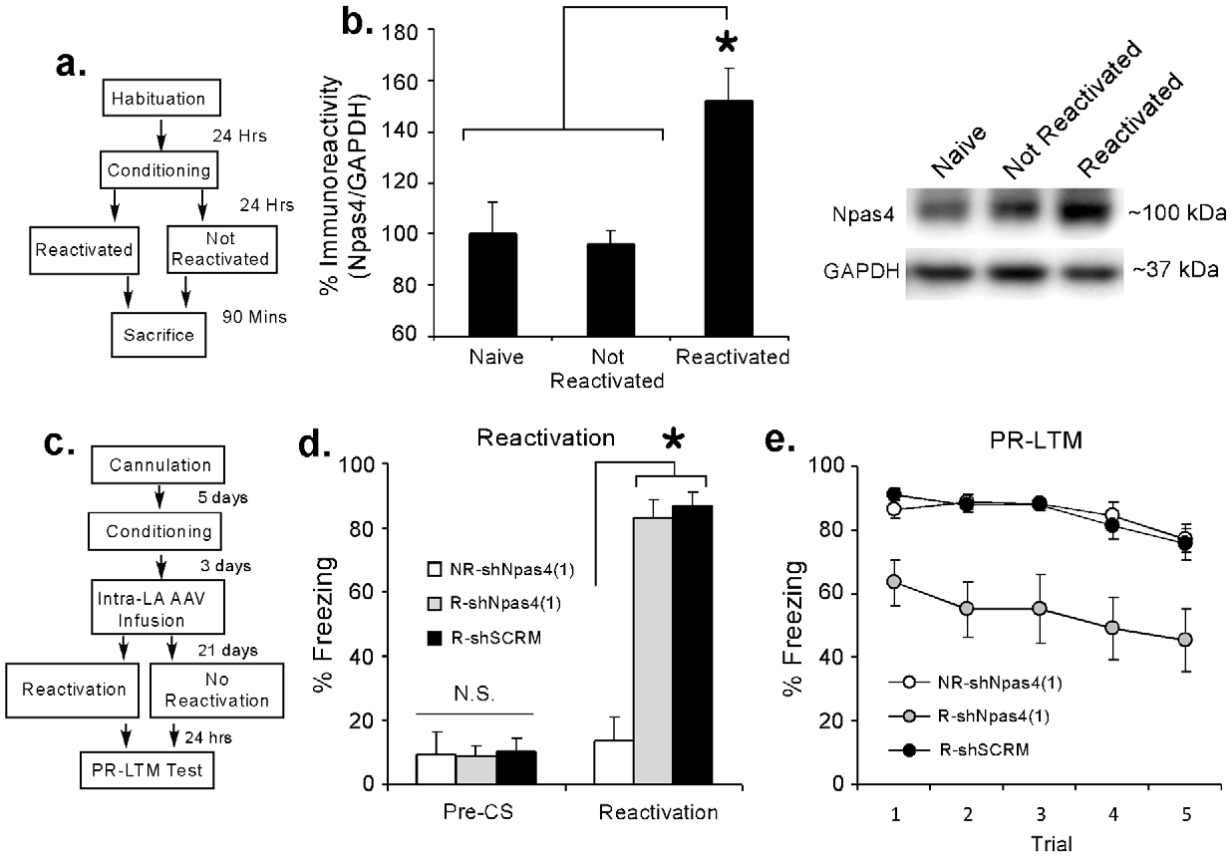
Knockdown of Npas4 in the LA impairs retention of a fear memory after retrieval. (A) Schematic of the behavioral protocol. Rats were habituated and trained and given either a reactivation or ‘no-reactivation’ session followed by sacrifice 90 min later. (B) Western blot analysis of Npas4 protein in the LA following fear memory retrieval. Rats were sacrificed 90 min following either retrieval of a fear memory (Reactivation; n = 8), exposure to the testing chamber without memory retrieval (No Reactivation; n = 8), or no stimulation (Naïve; n = 8). *p<0.05 relative to the other groups. Representative Western blots are shown in the inset. (C) Schematic of the behavioral protocol. Rats were trained followed 3 days later by intra-LA infusion of AAV- shNpas4(1) or AAV-shSCRM. Twenty-one days later, rats were placed in the testing chamber and given either a reactivation or ‘no-reactivation’ session followed by a test of post-reactivation LTM (PR-LTM) ∼24 hrs later. (D) Memory reactivation scores during the pre-CS and Reactivation periods for NR-shNpas4(1) (n = 6), R-Npas4(1) (n = 6), and R-shSCRM (n = 6) groups. *p<0.05 relative to the NR-shNpas4(1) group. (E) PR-LTM scores for NR-shNpas4(1), R-Npas4(1), and R-shSCRM groups across each trial.

In our first experiment, we used Western blotting to ask whether retrieval of an auditory fear memory regulates the expression of Npas4 protein in the LA. Rats were given auditory fear conditioning consisting of 2 presentations of a 20 sec, 5 kHz, 75 dB tone that co-terminated with a 1 sec, 2 mA foot shock. The following day, rats were placed in the testing chamber and were exposed to either a single tone presentation, to serve as a memory reactivation trial, or to no tone presentation to serve as a ‘no reactivation’ control. All rats were then sacrificed 90 min later (Figure 5a). Retrieval of an auditory fear memory led to a significant increase in Npas4 expression in the LA, F(2,21)  = 8.52, p<0.01. Duncan's post-hoc t-tests revealed that the Reactivated group exhibited significantly more Npas4 expression relative to either the Non-reactivated or Naïve groups (p<0.05), while no significant differences were observed between the Naïve and Non-reactivated groups (Figure 5b).

#### Knockdown of Npas4 in the LA impairs retention of a fear memory after retrieval

In our second experiment, we examined the effect of Npas4 knockdown in the LA following retrieval of an auditory fear memory. Rats were given auditory fear conditioning consisting of 2 presentations of a 20 sec, 5 kHz, 75 dB tone that co-terminated with a 1 sec, 2 mA foot shock followed 3 days later by intra-LA infusion of AAV-shNpas4(1) or AAV-shSCRM (1 µl/side). Twenty-one days later, rats were placed in the testing chamber and exposed to a single tone presentation to serve as a memory reactivation trial. An additional group of rats infused with AAV-shNpas4(1) was placed in the testing chamber for an equivalent amount of time, but received no tone presentation to serve as a ‘no reactivation’ control. All rats were then given a test of post-reactivation LTM (PR-LTM) ∼24 hrs later (Figure 5c).

No significant differences were detected in pre-CS freezing between the non-reactivated (NR)-shNpas4(1), reactivated (R)-shNpas4(1), and R-shSCRM groups, F(2,15)  = 0.01, p<0.05. During the reactivation period, however, R-shNpas4(1) and R-shSCRM groups exhibited high levels of freezing, while the NR-shNpas4(1) group did not, F(2,15)  = 33.81, p<0.01. Duncan's post-hoc t-tests revealed a significant difference between the NR-shNpas4(1) group and the R-shNpas4(1) and R-shSCRM groups (p<0.05), while no significant difference was observed between the R-shNpas4(1) and R-shSCRM groups (p>0.05; Figure 5d). During the PR-LTM test, however, the R-shNpas4(1) group exhibited impaired fear memory retention relative to the other groups (Figure 5e). The ANOVA revealed significant effects of group [F(2,14)  = 9.06, p<0.01] and trial [F(4,60)  = 5.06, p<0.01], but no significant group by trial interaction [F(8,60)  = 0.38, p>0.05].

Thus, knockdown of Npas4 in the LA impairs fear memory retention following retrieval, consistent with the notion that signaling via Npas4 is critical for fear memory reconsolidation.

## Discussion

While CREB-dependent transcription has been widely implicated in memory formation [2], [3], [4], including the consolidation and reconsolidation of fear memory memories [16], [17], [18], considerably less is known about the role of other transcription factors in each of these processes. In the present study, we provide the first evidence that the transcription factor Npas4 is required for mammalian memory formation. We show that Npas4 mRNA and protein are regulated in the LA in a learning-dependent manner following both auditory Pavlovian fear conditioning and retrieval of an auditory fear memory. Further, viral-mediated knockdown of Npas4 protein in the LA via RNAi impairs fear memory formation and retention of a fear memory following retrieval, while innate fear and the expression of fear memory are not affected.

Recently, Npas4 has been implicated in regulating a transcriptional program that participates in homeostatic plasticity where it induces inhibitory synapse development [10]. In that study, depletion of Npas4 via RNAi in dissociated hippocampal cell cultures promoted a decrease in inhibitory synapse number and a corresponding decrease in mIPSC amplitude and frequency, while having no effect on the number of excitatory synapses. Further, the decrease in inhibitory synapse number was observed to be associated with an increase in the frequency of mEPSCs [10]. These findings suggest that Npas4 is critical for regulating the balance of excitation and inhibition in neural circuits, and raise the interesting question of how knockdown of Npas4 *in vivo* might affect memory formation. Would depletion of Npas4 enhance memory formation due to a lack of inhibition of excitatory neural activity, or would knockdown of Npas4 impair memory formation? Our findings in the fear memory system suggest the latter. Further, Lin et al (2008) noted that mice that lack Npas4 exhibit an anxious and hyperactive phenotype. In our experiments, we observed that both innate fear in the elevated plus maze and the expression of a previously acquired fear memory are unaffected following treatment with AAV-shNpas4. These observations suggest that the effects of Npas4 depletion in the LA on fear memory are not secondary to alterations in general levels of anxiety or activity. Additional experiments will be required to appreciate how depletion of Npas4 affects neurophysiological properties of LA neurons and both excitatory and inhibitory synaptic plasticity within the LA.

The downstream genes that Npas4 targets to promote memory formation and retention of a fear memory after retrieval are at present unknown. A recent study has suggested that Npas4 regulates a wide variety of genes that are known to be critical for synaptic plasticity, including those that encode channel proteins, protein kinases and phosphatases, and proteins involved in synaptic trafficking and receptor endocytosis [10]. Further, it has been shown that Npas4 controls a transcriptional program which includes brain derived neurotrophic factor (BDNF) [10], a gene that has previously been shown to be essential for synaptic plasticity and memory formation, including fear memory formation [19]. Interestingly, BDNF is not only important for learning and memory, but also for inhibitory synapse development [20], [21], [22], [23]. The link between Npas4 and BDNF in amygdala-dependent fear conditioning and synaptic plasticity will require further study.

In summary, this is the first study to show a functional role for Npas4 in learning and memory. Our findings expand nicely upon previous work that has documented a role for Npas4 in inhibitory synapse development and synaptic plasticity, and further contribute to our understanding of the cellular and molecular mechanisms of fear memory formation within the LA.

## Materials and Methods

### Subjects

Adult male Sprague Dawley rats (Harlan) were housed individually in plastic cages and maintained on a 12 hr light/dark cycle. Food and water were provided *ad libitum*.

### cDNA constructs

Rat Npas4 (GenBank Accession No. NM_153626) cDNA was obtained by PCR amplification with gene specific primers (Forward primer: CCGCTCG-AGATGGACCGATCCACCAAGGGC; Reverse primer: GGAATTCGAAACGTTG-GTTCCCCTCCAC) from oligo dT primed rat brain cDNA and cloned into the XhoI and EcoRI sites of the pdsRedN-N1 (Clontech). Short-hairpin oligonucleotides designed to target Npas4 mRNA for degradation were annealed and cloned into SapI/XbaI sites of the AAV-GFP plasmid, a modified pCMV-MCS AAV vector [14]. The oligonucleotides sequences were as follows: shNpas4-1FP, TTTGTGAACTGGTTAGTGGAT-CTGGGACTTCCTGTCATCCCAGATCCACTAACCAGTTCAATTTTT; shNpas4-1RP, CTAGAAAAATTGAACTGGTTAGTGGATCTGGGATGACAGGAAGTC-CCAGATCCACTAACCAGTTCAC; shNpas4-5FP; TTTGATCTGCTGGATGAG-ACGATCTATCTTCCTG-TCAATAGATCGTCTCATCCAGCAGATATTTTT; shNpas4-5RP, CTAGAAAAATATCTGCTGGATGAGACGATCTATTGACAGGAA-GATAGATCGTCTCATCCAGCAGATC. shRNAs were designed under the guidelines previously outlined [24]. All constructs were verified by sequencing.

### Virus production and purification

AAV was produced using a triple-transfection, helper-free method, as previously described [14], [25].

### *In vitro* Npas4 knockdown

HEK293 cells (ATCC, Cat# crl 1573) were seeded in a 24 well cell culture plate at a density of ∼5×103 per well. When the cells reached ∼40–50% confluency, they were transfected with the plasmid dsRed1-N1 expressing rat Npas4 as a fusion protein with red fluorescent protein (RFP) and shNpas4(1) or shNpas4(5) or shSCRM plasmids in a 1∶1 ratio complexed with Liptofectamine 2000 (Invitrogen) transfection reagent. Ninety-six hours later, the cells were analyzed by fluorescence microcopy for an assessment of transfection efficiencies of the green fluorescent protein (GFP) expressing AAV-shRNA plasmids and for a qualitative assessment of Npas4-RFP depletion. For a quantitative assessment of the efficiency of Npas4 depletion, cells were transfected with the appropriate plasmids as above. Ninety-six hours later cells were harvested, RNA was purified and converted to cDNA, and qRT-PCR was performed to detect changes in Npas4 expression between shRNA transfected cells vs. those transfected with an AAV-shSCRM. To ensure these results were not a result of a bias in transfection efficiency of the Npas4-RFP plasmid, qRT-PCR data was normalized to the neomycin-resistance gene which is expressed solely from the pdsRed1-N1 plasmid [26].

### Quantitative real-time PCR (qRT-PCR) experiments

To examine the time course of Npas4 mRNA regulation in the LA, rats were habituated to handling for 4 days prior to receiving three conditioning trials consisting of a 20 sec, 5 kHz, 75 dB tone that co-terminated with a 1 sec, 0.5 mA foot shock [ITI  = 120 sec]. “Naïve” control rats were handled and sacrificed without further stimulation. To examine whether Npas4 regulation in the LA is specific to fear conditioning, rats were handled and trained as above. “Immediate Shock” control rats were placed in the conditioning chamber and immediately given three 0.5 mA foot shocks and removed from the training chamber, a procedure that does not support fear learning [27]. “Naïve” control rats were handled and sacrificed without further stimulation. In a separate experiment, rats were handled and habituated to the conditioning chamber for 4 days prior to training (as above). “Tone Only” control rats were exposed to three tones (20 sec, 5 kHz, 75 dB) without receiving shocks. “Box” rats were exposed to the conditioning chamber with no further stimulation. At the appropriate time, rats were anesthetized with CO_2_ and decapitated. Brains were removed, frozen on dry ice, and stored at −80°C.

The LA was microdissected and prepared for qRT-PCR as previously described [13]. Npas4 and GAPDH QuantiTect PCR primers were purchased from Qiagen. All qRT-PCR experiments were run in triplicate and relative gene concentrations were normalized against GAPDH levels. Data were analyzed using ANOVA and Duncan's post-hoc tests. All qRT-PCR data are represented as the average threshold cycle (Ct) difference values for each group after normalization to GAPDH, with the error bars representing the standard error of the mean for each group. Note that average fold change  = 2^(average Ct difference value)^.

### Western blotting

For fear acquisition experiments, rats were exposed to fear conditioning, immediate shock, tone alone, or naïve conditions as described above. For fear reactivation experiments, rats were given auditory fear conditioning as described above. The following day, rats were placed in the testing chamber and were exposed to either a single tone presentation (20 sec, 5 kHz, 75 dB), to serve as a memory reactivation trial, or to no tone presentation to serve as a ‘no reactivation’ control. Ninety min following either fear conditioning or reactivation of a fear memory, rats were euthanized with chloral hydrate (600 mg/kg; i.p.) and brains were frozen at −80°C. Tissue preparation and Western blotting were performed as previously described [28]. Western blots were blocked in TTBS buffer (50 mM Tris-HCl, pH 7.5, 150 mM NaCl, and 0.05% Tween 20) with 5% dry milk and then incubated with an anti-Npas4 antibody (1∶500; generous gift from Mehrdad Shamloo), anti-Arc antibody (1∶1K; Santa Cruz cat# sc-17839), or anti-c-Fos antibody (1∶1K; Santa Cruz cat# sc-52). Blots were then incubated with 1∶30K (Npas4) or 1∶20K (Arc and c-Fos) anti-rabbit IgG conjugated to horseradish peroxidase (Cell Signaling) and developed using West Dura chemiluminescent substrate (Pierce). Densitometry was conducted using Image J software. Optical densities were normalized to GAPDH protein (Abcam). Data were normalized to the average value of naïve controls and analyzed using ANOVA.

### Cannulation & AAV infusions

Surgical procedures were conducted as described previously [28]. Under a mixture of Ketamine (100 mg/kg) and Xylazine (6.0 mg/kg) anesthesia, rats were implanted bilaterally with 26-gauge stainless steel guide cannulas (Plastics One) aimed at the LA [Bregma -3.2 AP, ±5.0 ML, -8.0 DV]. Guide cannulas were fixed to screws in the skull using a mixture of acrylic and dental cement, and a 31-gauge dummy cannula was inserted into each guide to prevent clogging. Rats were given Buprenex (0.2 mg/kg) as an analgesic and given at least five days to recover prior to experimental procedures. All procedures were conducted in accordance to the National Institutes of Health *Guide for the Care and Use of Experimental Animals* and were approved by the Yale University Institutional Animal Care and Use Committee (Protocol #2010-10801; Animal Assurance Number A3230-01). On the day of infusions, dummy cannulas were removed and 33 gauge infusion cannulas which extended 2 mm below the guide cannula were inserted and 1 µl of AAV-shNpas4 or AAV-shSCRM was infused at a rate of 60 nl/min using an infusion pump. Following infusion, infusion cannulas remained implanted for an additional 10 min to allow diffusion of the virus from the infusion site. Behavioral training or testing began 21 days following viral infusion.

### Elevated plus maze

Prior to fear conditioning, a portion of the rats that had received intra-LA infusion of AAV-shNpas4 or AAV-shSCRM were tested in the elevated plus maze (EPM) to assess the effect of Npas4 knockdown on innate fear responses. The EPM was performed essentially as previously described [29]. Briefly, each rat was placed at the center of the maze, and the time it spent in the open arms and the numbers of entries into either the open or closed arms was measured for a period of 5 min. The task was carried out in a small, dimly lit room (100LUX).

### Fear conditioning experiments

Rats received intra-LA infusion of AAV-shNpas4 or AAV-shSCRM and were exposed to a single conditioning trial consisting of a 20 sec, 5 kHz, 75 dB tone that co-terminated with a 1 sec, 2 mA foot shock. Conditioning took place 21 days following viral infusion (or 22 days, if tested in the EPM). Testing for long-term memory (LTM) of conditioned fear was performed in a distinct environment 24 hours after training. For the retention test, rats were exposed to 10 conditioned stimulus tones (5 kHz, 75 dB, 30 sec). For behavioral experiments examining the effect of Npas4 knockdown retention of auditory fear conditioning, conditioned rats received intra-LA infusion of AAV-shNpas4 or AAV-shSCRM 2 days after training and were tested for retention of auditory fear conditioning 21 days later. For behavioral experiments examining the effect of Npas4 knockdown on retention of auditory fear conditioning following retrieval, conditioned rats received intra-LA infusion of AAV-shNpas4 or AAV-shSCRM 3 days after training and were given a memory reactivation trial (or no reactivation trial) 21 days later consisting of a single presentation of a 5 kHz, 75 dB, 20 sec tone. The following day, all rats received a post-reactivation (PR)-LTM (PR-LTM) test consisting of presentation of 5 tones 5 kHz, 75 dB, 20. For each behavioral experiment, total seconds freezing during the CS presentations was scored for each rat, and this number was expressed as a percentage of the total CS presentation time. All data were analyzed with ANOVA and Duncan's post-hoc t-tests.

### Imaging viral infection *in vivo*

To ascertain the location and spread of the viral infusions after each behavioral experiment, rats were perfused and brains were sectioned on a freezing sliding microtome. Sections (80 µM) were wet mounted on microscope slides and native GFP fluorescence was imaged using an Olympus BX51 microscope. Only rats with bilateral transfections confined to the borders of the LA were included in the behavioral analysis.
